# Associations among left ventricular systolic function, tachycardia, and cardiac preload in septic patients

**DOI:** 10.1186/s13613-017-0240-2

**Published:** 2017-02-17

**Authors:** Michael J. Lanspa, Sajid Shahul, Andrew Hersh, Emily L. Wilson, Troy D. Olsen, Eliotte L. Hirshberg, Colin K. Grissom, Samuel M. Brown

**Affiliations:** 1 Critical Care Echocardiography Service, Intermountain Medical Center, 5121 S Cottonwood St, Murray, UT 84157 USA; 2 Division of Pulmonary and Critical Care Medicine, University of Utah, 30 N 1900 E, 701 Wintrobe, Salt Lake City, UT 84132 USA; 3 Department of Anesthesia, Critical Care, and Pain Medicine, Beth Israel Deaconess Medical Center, Boston, MA USA; 4 Department of Anesthesia and Critical Care, University of Chicago, 5841 South Maryland Avenue, Chicago, IL 60637 USA; 5 Division of Pediatric Critical Care, Department of Pediatrics, University of Utah, 295 Chipeta Way, Salt Lake City, UT 84108 USA

**Keywords:** Strain, Echocardiography, Preload, Septic cardiomyopathy, Tachycardia

## Abstract

**Background:**

In sepsis, tachycardia may indicate low preload, adrenergic stimulation, or both. Adrenergic overstimulation is associated with septic cardiomyopathy. We sought to determine whether tachycardia was associated with left ventricular longitudinal strain, a measure of cardiac dysfunction. We hypothesized an association would primarily exist in patients with high preload.

**Methods:**

We prospectively observed septic patients admitted to three study ICUs, who underwent early transthoracic echocardiography. We measured longitudinal strain using speckle tracking echocardiography and estimated preload status with an echocardiographic surrogate (*E*/*e*′). We assessed correlation between strain and heart rate in patients with low preload (*E*/*e*′ < 8), intermediate preload (*E*/*e*′ 8–14), and high preload (*E*/*e*′ > 14), adjusting for disease severity and vasopressor dependence.

**Results:**

We studied 452 patients, of whom 298 had both measurable strain and preload. Abnormal strain (defined as >−17%) was present in 54%. Patients with abnormal strain had higher heart rates (100 vs. 93 beat/min, *p* = 0.001). After adjusting for vasopressor dependence, disease severity, and cardiac preload, we observed an association between heart rate and longitudinal strain (*β* = 0.05, *p* = 0.003). This association persisted among patients with high preload (*β* = 0.07, *p* = 0.016) and in patients with shock (*β* = 0.07, *p* = 0.01), but was absent in patients with low or intermediate preload and those not in shock.

**Conclusions:**

Tachycardia is associated with abnormal left ventricular strain in septic patients with high preload. This association was not apparent in patients with low or intermediate preload.

**Electronic supplementary material:**

The online version of this article (doi:10.1186/s13613-017-0240-2) contains supplementary material, which is available to authorized users.

## Background

Sepsis is a common and often lethal state of extreme disruption of homeostasis in the face of severe infection [1, 2]. Administration of exogenous catecholamines to maintain adequate arterial pressure is the cornerstone of current management of septic shock, despite the 20-fold increase in endogenous catecholamine levels observed in patients with septic shock [3] and increasing evidence for catecholamine toxicity as an important factor in septic shock physiology [4, 5]. This hyperadrenergic state, in combination with excess cytokine production, results in a spectrum of myocardial injury often grouped under the general category of septic cardiomyopathy. Septic cardiomyopathy is remarkably common in sepsis, despite a historical belief that sepsis was primarily or exclusively a hyperdynamic state [5, 6]. Exogenous catecholamine therapy can improve myocardial contraction and is typically used to treat septic cardiomyopathy. However, catecholamine administration may paradoxically worsen cardiac function [5]. In animals, infusion of epinephrine into coronary arteries induces a cardiomyopathy [7]. In humans undergoing routine cardiac stress testing, dobutamine infusions can directly induce cardiomyopathy [8–10].

Catecholamines are a key mediator of baroreflex function, by which the autonomic nervous system optimizes cardiac output and blood pressure through adjustments in heart rate, contractility, and vascular tone. Early in sepsis, tachycardia may merely reflect appropriate baroreflex activity [11]. In septic shock, however, the baroreflex system often malfunctions. After adequate volume expansion, persistent tachycardia in sepsis likely reflects an inappropriately hyperadrenergic state. In this respect, persistent tachycardia in septic shock may be similar to the excess tachycardia observed in stress cardiomyopathy [12]. Such persistent tachycardia is an independent risk factor for mortality in patients with sepsis [13–15]. The relationship between higher heart rate and poor outcome in established septic shock extends even to relatively low (<60–80/min) heart rates [16].

Historically, cardiac function in sepsis has been assessed primarily by left ventricular ejection fraction (LVEF). However, LVEF is highly load dependent and therefore less reliable in assessment of cardiac function in states of low preload or low afterload [17–21]. In the initial phase of septic shock, hypovolemia can occur with decreased preload and afterload related to increased capillary leak [22] and low vascular resistance [23, 24]. The development of speckle tracking echocardiography has made possible the measurement of ventricular longitudinal strain, a measure of deformation of the ventricular wall [25, 26]. Strain imaging has been demonstrated to detect subclinical myocardial dysfunction in animal models [27, 28], vigorously exercising healthy adults [29, 30], and a number of disease states, including reduced preload or afterload states such as sepsis [26, 31–36].

Given the improved ability to detect septic cardiomyopathy provided by the development of LV strain imaging techniques, we have a new opportunity to investigate the relationship between hyperadrenergic states in sepsis and septic cardiomyopathy. We hypothesized that in septic patients with adequate or increased preload, tachycardia would be associated with worse ventricular strain, suggesting the possibility that hyperadrenergism, manifested by increased heart rate in the absence of low preload, is associated with septic cardiomyopathy, while tachycardia in low-preload states would not be associated with impaired LV strain.

## Methods

Study Design: This prospective, observational study was conducted at three intensive care units (ICUs) at two study hospitals, Intermountain Medical Center and Beth Israel Deaconess Medical Center. In these ICUs, transthoracic echocardiography (TTE) is routinely performed on patients with sepsis or septic shock at the time of ICU admission. The protocol was approved by the Intermountain Healthcare Institutional Review Board with a waiver of informed consent and by the Beth Israel Deaconess Medical Center Institutional Review Board with oral informed consent.

### Patients

We screened patients between October 2012 and November 2015 admitted with severe sepsis or septic shock defined by the then-current 1992 American College of Chest Physicians/Society of Critical Care Medicine consensus criteria [37], and operationalized by recent large sepsis trials [38–40]. Patients met criteria for inclusion if they (1) were at least 18 years of age, (2) had clinically suspected infection, (3) had two or more systemic inflammatory response syndrome criteria, and (4) had either septic shock (systolic blood pressure <90 mmHg despite an intravenous fluid challenge of ≥20 ml/kg or infusion of any dose of vasopressor medications) or severe sepsis (defined in this study as serum lactate ≥4 mmol/L). An unrelated study of definitions of diastolic dysfunction [41] included 129 (28.5%) of the patient population we analyzed for the present study [41].

### Clinical data

We calculated Acute Physiology and Chronic Health Evaluation, 2nd version (APACHE II) [42] and Sequential Organ Failure Assessment (SOFA) [43] scores at ICU admission for all patients. We determined receipt of mechanical ventilation and the vasopressor (norepinephrine, epinephrine, dopamine, phenylephrine, and vasopressin) infusion rate at the time the echocardiogram was obtained. We converted the sum of vasopressor infusion rates to norepinephrine-equivalent rates according to standard equivalencies [44]. We defined presence of shock based on vasopressor receipt at the time of echo [45]. We assessed both inpatient and 28-day mortality and calculated organ-failure-free days out of 14 days for the cardiovascular, coagulation, hepatic, and renal components of the SOFA score. We recorded serum troponin in patients who had a clinically obtained serum troponin closest to the time of the echocardiogram, within 24 h. In patients enrolled in the Intermountain ICUs, we also recorded the amount of intravenous fluid administered in the 6 h preceding the echocardiogram.

### Transthoracic echocardiography

Transthoracic echocardiograms (TTEs) were performed by a cardiac sonographer or a physician echocardiographer, using a Philips iE-33 or CX-50 (Philips Medical Systems, Bothell, WA). Patients were excluded if their TTE occurred more than 24 h after ICU admission or if the image quality was so poor as to be uninterpretable. All echo readers were blinded to clinical outcomes, and all final interpretations were performed by Level II echocardiographers who are testamurs of the National Board of Echocardiography Adult Comprehensive Exam. Longitudinal strain was measured independently from preload assessment, to avoid possible bias. We used the Image-Arena platform (TomTec Imaging Systems, Unterschleissheim, Germany) to perform semiautomated speckle tracking for left ventricular longitudinal strain. We selected standard apical four-chamber views for strain analysis. All strain analyses were performed by advanced cardiac sonographers or physicians who had already performed >100 h of speckle tracking analysis. We selected the best available single cardiac cycle with regard to image quality and measured longitudinal strain of the endocardium. We rejected images due to poor image quality if we could not track two or more adjacent segments in the apical four-chamber view. We defined abnormal strain as greater than −17% (higher numbers are worse) in accordance with previously published literature describing patients with septic shock [20, 46]. Because central venous pressure is no longer widely measured in the study ICUs, we defined cardiac preload using the ratio of early diastolic septal mitral inflow velocity to early diastolic mitral annulus velocity (*E*/*e*′) to assess left ventricular preload [41, 47, 48]. We defined a low-preload state as an *E*/*e*′ < 8, a high-preload state as >14, and an intermediate-preload state as 8–14 [49, 50]. In the subset of patients from the Intermountain ICUs, we formally categorized diastolic function in accordance with the 2016 American Society of Echocardiography guidelines [51].

### Statistical analysis

Our prespecified primary analysis was a linear regression of LV longitudinal strain on heart rate, while controlling for vasopressor infusion rate, preload (*E*/*e*′), and admission APACHE II. To better understand the relevance of preload in this analysis, we secondarily fit separate regression models (controlling for vasopressor infusion rate and APACHE II) for patients with low preload, intermediate preload, or high preload.

For purposes of description, we compared various patient characteristics and clinical outcomes according to preload status, shock status (receipt of vasopressors), and whether they met criteria for abnormal strain (>−17%). Low-, intermediate-, and high-preload patients were analyzed using Kruskal–Wallis tests for comparisons of central tendencies, while Wilcoxon rank sum tests were used to compare patients with and without shock, as well as those with and without abnormal strain. Fisher’s exact tests were used to compare proportions for all three analyses. Statistical analyses were performed using the R Statistical Package, version 3.0.2 [52].

## Results

**Fig. 1:**
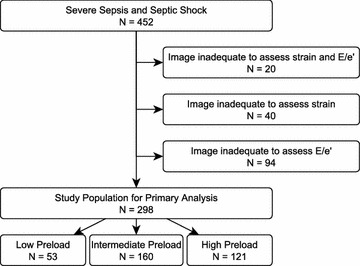
Study inclusion and exclusion. *E*/*e*′: ratio of early diastolic mitral inflow blood velocity to early diastolic mitral annulus tissue velocity, a surrogate for ventricular preload

Our study population comprised 452 patients, of whom 392 (87%) had adequate echocardiographic image quality to measure longitudinal strain and 338 (75%) had a measureable *E*/*e*′ (Fig. 1). Seventy-eight percent of patients also met Sepsis-3 criteria [45]. We performed our primary analysis on the 298 patients who had both strain and *E*/*e*′ available. Echocardiograms were obtained quickly: median 2.3 h after ICU admission. Patient characteristics are displayed in Table 1. The patients had a median SOFA score of 9 (IQR 6–12) and an APACHE II score of 25 (IQR 18–23), with an overall 28-day mortality of 23%. At the time of the echocardiogram, 31% patients were undergoing mechanical ventilation and 39% were in shock. Troponin was elevated (≥0.5 ng/mL) in 41% of patients (median 0.05 ng/mL, IQR 0.02–0.18). Patients with elevated troponin had worse strain (−14.6 vs. −16.9, *p* = 0.04), but no difference in heart rate (97 vs. 102, *p* = 0.18). Fifty-four percent of the patients in the primary analysis had abnormal strain, and 36% had high preload (*E*/*e*′ > 14). Patients with abnormal strain had higher heart rates (100 vs. 93 beat/min, *p* = 0.001). We noted no difference between patients with shock and without shock in regard to heart rate (99 vs. 95, *p* = 0.88), ejection fraction (61% for both, *p* = 0.22), or strain (−16 vs. −17%, *p* = 0.93). Patients in shock had a lower *E*/*e*′ (10.6, IQR 8.3–13.9) than those without shock (13.2, IQR 9.6–18.6, *p* < 0.001).

**Table 1:** Patient characteristics, categorized by preload

Variable [median (IQR) or *N* (%)]	Overall *N* = 452	Preload (*N* = 338)			*p* value
		*E*/*e*′ < 8 *N* = 57	8 ≤ *E*/*e*′ ≤ 14 *N* = 160	*E*/*e*′ > 14 *N* = 121	
Cohort details					
Female	240 (53%)	22 (39%)	78 (49%)	89 (74%)	<0.001
Age, years	65 (54–75)	59 (46–71)	64 (52–73)	71 (62–78)	<0.001
Body mass index	28 (24–34)	26 (24–31)	28 (24–33)	29 (25–33)	0.44
APACHE II	25 (18–33)	29 (18–36)	24 (16–33)	24 (18–29)	0.10
SOFA	9 (6–12)	9 (6–13)	9 (6–12)	8 (6–10)	0.09
MAP, mm Hg	69 (61–77)	69 (65–77)	69 (61–76)	70 (60–76)	0.61
Receiving vasopressors	175 (39%)	31 (54%)	74 (46%)	35 (29%)	0.001
Norepinephrine-equivalent dose, mcg/kg/min^a^	0.14 (0.07–0.30)	0.17 (0.09–0.29)	0.15 (0.06–0.34)	0.09 (0.06–0.22)	0.23
Mechanically ventilated	138 (31%)	31 (54%)	44 (28%)	24 (20%)	<0.001
PiO_2_/FiO_2_ ratio, mm Hg	234 (160–335)	223 (145–316)	248 (165–365)	250 (185–360)	0.66
Serum lactate, mmol/dL	2.3 (1.4–3.8)	2.6 (1.5–4.0)	2.4 (1.5–3.9)	1.8 (1.2–3.5)	0.08
Heart rate, BPM	97 (83–112)	100 (86–114)	98 (83–112)	93 (78–106)	0.08
Fluid (6 h prior to echo), L^b^	3.0 (1.2–4.0)	3.0 (1.9–4.8)	3.4 (2.0–5.0)	2.0 (1.0–4.0)	0.004
Echocardiographic parameters					
Ejection fraction, %	61 (52–69)	61 (50–70)	62 (56–67)	62.8 (52–70)	0.69
Strain, %	−17 (−20 to −12)	−16 (−20 to 11)	−17 (−20 to 13)	−16 (−20 to 13)	0.38
*E*/*e*′	11.9 (8.9–16.3)	6.7 (5.9–7.4)	10.5 (9.4–12.2)	18.5 (15.7–21.8)	<0.001
Stroke volume, mL	56 (45–70)	48 (37–65)	61 (47–72)	60 (48–75)	0.015
Diastolic function^b^					<0.001
Grade 0 (normal)	197 (49%)	26 (65%)	106 (72%)	16 (14%)	
Grade 1	23 (6%)	5 (13%)	12 (8%)	2 (2%)	
Grade 2	43 (11%)	1 (2%)	0 (0%)	40 (34%)	
Grade 3	16 (4%)	1 (2%)	1 (1%)	12 (10%)	
Indeterminate	119 (30%)	7 (18%)	27 (18%)	48 (41%)	
Clinical outcomes					
Inpatient mortality	87 (19%)	14 (25%)	28 (18%)	21 (17%)	0.46
28-Day mortality	103 (23%)	14 (25%)	33 (21%)	29 (24%)	0.73
OFFD cardiovascular to day 14	13 (9–13)	12 (7–13)	13 (9–13)	13 (10–13)	0.08
OFFD coagulation to day 14	14 (13–14)	14 (12–14)	14 (13–14)	14 (13–14)	0.98
OFFD hepatic to day 14	14 (12–14)	14 (10–14)	14 (12–14)	14 (13–14)	0.50
OFFD renal to day 14	13 (11–14)	13 (9–14)	13 (12–14)	13 (12–14)	0.39

Preload was defined according to the ratio of early diastolic mitral filling to early mitral annular tissue velocity (*E*/*e*′)

*APACHE* Acute Physiology and Chronic Health Evaluation, *SOFA* Sequential Organ Failure Assessment, *MAP* mean arterial pressure, *E*/*e*′ ratio of early diastolic mitral inflow blood velocity to early diastolic mitral annular tissue velocity. *OFFD* organ-failure-free days

^a^Among those receiving vasopressors

^b^These data were only collected in the 398 patients from Intermountain ICUs, 40 with low preload, 146 with intermediate preload, 118 with high preload

**Fig. 2:**
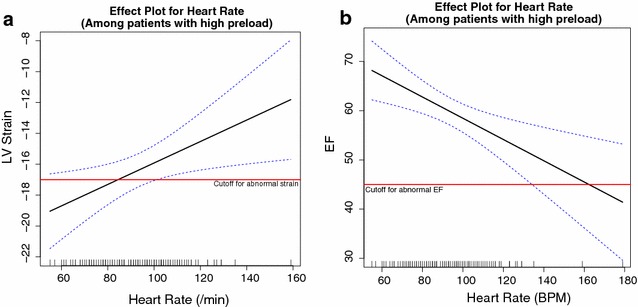
**a** Effect plot for heart rate and longitudinal strain; **b** effect plot for heart rate and ejection fraction among patients with high cardiac preload and controlling for vasopressor dose and APACHE II score. The plots depict thresholds for abnormal strain (−17%) and abnormal ejection fraction (45%) [46]

We observed no difference in heart rate among patients with low, intermediate, and high preload (median 100, 98, and 93 beat/min, *p* = 0.08). Patients with low preload had lower stroke volume, and patients with higher preload received less fluid (Table 1). After adjusting for vasopressor dosage, disease severity, and preload, we observed an association between heart rate and strain (*β* = 0.05, *p* = 0.003, Table 2). In the stratified models based on low, intermediate, or high preload, we observed among patients with high preload an association between heart rate and longitudinal strain after adjusting for vasopressor dosage and disease severity (*β* = 0.07, *p* = 0.016, Table 2; Fig. 2). This association was absent in patients with low preload (*p* = 0.28) or intermediate preload (*p* = 0.19). In an exploratory analysis, we evaluated the expected negative correlation between ejection fraction and strain (*r* = −0.40 in low preload, −0.31 in intermediate preload, and −0.60 in high preload). We observed an association between ejection fraction and heart rate in patients with high preload (*β* = −0.22, *p* = 0.002) that was absent in patients with low preload (*p* = 0.80) or intermediate preload (*p* = 0.87). We observed a similar association between strain and heart rate among patients with shock (*β* = 0.07, *p* = 0.01) that was absent in non-shock patients (Table 2). Additional clinical data on patients stratified by presence of shock are available (Additional file 1: Table S1).

**Table 2:** Multivariable linear regression for longitudinal LV strain among patients, among all patients, and stratified according to preload (*E*/*e*′) and according to presence of shock (receiving a vasopressor at the time of echocardiogram)

All patients with interpretable TTEs (*N* = 298)	Coefficient	*p* value
Heart rate	0.05	0.003
NEE during echo (per 0.01 mcg/kg/min increase)	0.02	0.17
APACHE II	0.03	0.37
*E*/*e*′	0.11	0.02
Low preload *E*/*e*′ < 8 (*N* = 53)		
Heart rate	−0.03	0.80
NEE during echo (per 0.01 mcg/kg/min increase)	0.02	0.90
APACHE II	0.12	0.56
Intermediate preload 8 ≤ *E*/*e*′ ≤ 14 (*N* = 135)		
Heart rate	−0.01	0.87
NEE during echo (per 0.01 mcg/kg/min increase)	0.005	0.86
APACHE II	0.09	0.33
High preload *E*/*e*′ > 14 (*N* = 110)		
Heart rate	−0.22	0.002
NEE during echo (per 0.01 mcg/kg/min increase)	0.02	0.87
APACHE II	−0.02	0.87
Non-shock (*N* = 174)		
Heart rate	0.04	0.10
APACHE II	0.06	0.20
*E*/*e*′	0.12	0.02
Shock (*N* = 124)		
Heart rate	0.07	0.01
NEE during echo (per 0.01 mcg/kg/min increase)	0.02	0.36
APACHE II	−0.003	0.96
*E*/*e*′	0.04	0.70

*APACHE II* Acute Physiology and Chronic Health Evaluation, version 2, *NEE* norepinephrine-equivalent dose. *TTE* transthoracic echocardiogram

In our secondary analysis of organ dysfunction, patients with normal LV strain had greater renal-failure-free days than patients with abnormal strain (14 vs. 13, *p* = 0.01). We found no difference in hospital mortality or in cardiovascular-, hepatic-, or coagulation-failure-free days between patients with normal strain versus patients with abnormal strain (Table 3).

**Table 3:** Clinical outcomes between normal and abnormal strain, stratified by preload status

Parameter	Abnormal strain (*n* = 214)	Normal strain (*n* = 178)	*p* value
Overall			
Hospital mortality	47 (22%)	31 (17%)	0.31
28-Day mortality	54 (25%)	36 (20%)	0.28
OFFD to day 14—cardiovascular	13 (9–13)	12 (8–13)	0.37
OFFD to day 14—coagulation	14 (13–14)	14 (13–14)	0.33
OFFD to day 14—hepatic	14 (13–14)	14 (12–14)	0.71
OFFD to day 14—renal	13 (10–14)	14 (13–14)	0.01

Low preload (*E*/*e*′ < 8)	*N* = 33	*N* = 20	
Hospital mortality	8 (24%)	5 (25%)	1.00
28-Day mortality	8 (24%)	4 (20%)	1.00
OFFD to day 14—cardiovascular	12 (9–13)	10 (7–13)	0.53
OFFD to day 14—coagulation	14 (13–14)	14 (11–14)	0.39
OFFD to day 14—hepatic	14 (13–14)	14 (3–14)	0.22
OFFD to day 14—renal	13 (9–14)	13 (11–14)	0.96

Intermediate preload (*E*/*e*′ 8–14)	*N* = 66	*N* = 69	
Hospital mortality	14 (21%)	12 (17%)	0.66
28-Day mortality	15 (23%)	15 (22%)	1.00
OFFD to day 14—cardiovascular	12 (9–13)	13 (8–13)	0.86
OFFD to day 14—coagulation	14 (11–14)	14 (13–14)	0.54
OFFD to day 14—hepatic	14 (11–14)	14 (12–14)	0.70
OFFD to day 14—renal	13 (9–14)	14 (13–14)	0.05

High preload (*E*/*e*′ > 14)	*N* = 63	*N* = 47	
Hospital mortality	13 (21%)	6 (13%)	0.32
28-Day mortality	18 (29%)	9 (19%)	0.27
OFFD to day 14—cardiovascular	13 (8–13)	13 (10–13)	0.97
OFFD to day 14—coagulation	14 (13–14)	14 (14–14)	0.14
OFFD to day 14—hepatic	14 (13–14)	14 (14–14)	0.13
OFFD to day 14—renal	13 (11–14)	14 (13–14)	0.22

Abnormal strain is defined as >−17% [46]

*OFFD* organ-failure-free days

## Discussion

In a large, multicenter cohort of septic patients undergoing echocardiography, tachycardia was associated with worsened ventricular strain. This association persisted after adjusting for preload, vasopressor dose, and severity of illness. This association appeared to be restricted to patients with high preload, as estimated by an *E*/*e*′ > 14, a threshold normally associated with left arterial hypertension. The observation that tachycardia in sepsis is associated with worsened clinical outcomes is well established in the literature [14, 16, 53]. However, prior literature has not distinguished between tachycardia that likely represents a hyperadrenergic state, which may contribute to septic cardiomyopathy from tachycardia that likely reflects the adaptive response to low preload. Elevated heart rate may not be a simple surrogate for increased disease severity and increased receipt of vasopressors [16]. Our study, which controlled for severity of illness and vasopressor infusion rates, demonstrates the importance of assessing cardiac preload when evaluating the implication of tachycardia in sepsis. The association between impaired strain and tachycardia adds further evidence to the evolving literature associating high adrenergic tone with septic cardiomyopathy [16, 28, 54]. Some controversy exists regarding treatment of tachycardic septic patients with beta blocker therapy [55]. While our findings are too preliminary to be used to identify patients who might benefit from beta-blockade in sepsis, speckle tracking echocardiography may be of use in designing future investigations of beta-blockade in sepsis.

One challenge in discussing septic cardiomyopathy is that the term has been used to describe different pathophysiologic states. Septic cardiomyopathy may refer to (a) decreased ejection fraction or stroke volume (which can occur with either high or low ejection fraction) [56], (b) new or worsened diastolic dysfunction [57, 58], and (c) cardiomyocyte dysfunction (including mitochondriopathy, calcium handling, apoptosis, and hibernation) [59]. Some definitions of septic cardiomyopathy may thus be simple surrogate measures for cardiac filling pressures or the severity of the underlying shock, while others reflect intrinsic cardiomyocyte dysfunction. Our data suggest that when controlling for cardiac preload, a sensitive measure of myocardial dysfunction is associated with tachycardia, itself a useful surrogate for hyperadrenergia.

Our proportion of patients with septic cardiomyopathy compares similarly to other published cohorts [20, 46, 60]. The use of longitudinal LV strain as the measure of left ventricular systolic function has advantages over LVEF, the historical measure. Despite being the common method to assess ventricular systolic function, LVEF varies with loading conditions and heart rate, is poorly reproducible for different observers, and is likely inferior to strain in representing intrinsic cardiac systolic function [34]. Our prior work in this area demonstrated that ventricular strain was better associated with clinical measures of the adequacy of perfusion than LVEF [20]. However, on the basis of our stratified analysis, in patients with high preload, LVEF is associated with the degree of tachycardia. The correlation between strain and LVEF is moderate, suggesting that they measure different aspects of myocardial function. As demonstrated in the effect plots and expected on the basis of prior studies, abnormal strain is more common than abnormal LVEF. We hypothesize that prior work has not adequately distinguished between an elevated LVEF that reflects low cardiac preload from an elevated LVEF that reflects an absence of septic cardiomyopathy. By controlling for cardiac preload, we may have allowed a more accurate assessment of the association between tachycardia and ventricular systolic dysfunction.

We acknowledge that *E*/*e*′ is an imperfect surrogate for left ventricular preload or hypovolemia [61]. Age, mitral valve disease, pericardial disease, mechanical ventilation, and regional wall motion abnormality all may affect the accuracy of *E*/*e*′. Our regression models included age as a component of APACHE II score, and a sensitivity analysis including age as a separate covariate demonstrated age was not significantly associated with strain. Among critically ill patients, particularly an elevated *E*/*e*′ may be difficult to interpret. An increased *E*/*e*′ might reflect impaired myocardial diastolic function but could also result from hypervolemia with normal myocardial diastolic function. In addition, while *E*/*e*′ > 14 accurately identifies patients with high cardiac preload, *E*/*e*′ < 14 may be difficult to interpret [51]. While a low *E*/*e*′ is associated with lower filling pressures, a low *E*/*e*′ does not necessarily indicate hypovolemia. This large zone of uninterpretable values for *E*/*e*′ underlies the rejection of *E*/*e*′ as the sole measure of diastolic function in the ASE 2016 definitions, although use of the septal *E*/*e*′ as a sole measure of diastolic function has been studied in the critically ill [41]. In addition, we acknowledge that we employed septal *E*/*e*′ rather than the average of septal and lateral *E*/*e*′ due to image availability and based on previously published data suggesting adequate accuracy [48]. This may have made our measurements of *E*/*e*′ more difficult to compare to values published using the average of septal and lateral.

Alternative measures of assessing cardiac preload are available, although many have limitations, and there is no clinically available gold standard. In the contemporary ICU, it is exceedingly uncommon to place pulmonary artery catheters in septic patients to assess preload. Dynamic parameters such as pulse pressure variation, aortic velocity variation, or vena cava variation depend on passive mechanical ventilation, which is also uncommon in contemporary ICUs [62]. The response to a passive leg raise (or similar provocative maneuvers like the expiratory occlusion test) [63] may have been informative, but was unfortunately not performed at the time of the echocardiogram in study patients.

Future scientific inquiry in this field may benefit from a composite of several available surrogates of cardiac preload, including dynamic parameters, passive leg raise, *E*/*e*′, shock index, and central venous pressure. A composite of these measurements may outperform a single surrogate measurement [64]. Perhaps more important, future studies would benefit from protocolized timing of echocardiography in relationship to fluid administration, perhaps immediately after receipt of the initial 30 ml/kg volume expansion, and again immediately when surrogates of cardiac preload indicate that the patient is no longer fluid responsive. Most studies in this field, including the present study, have not dictated timing of echocardiography at a specific phase of sepsis resuscitation, resulting in unnecessary heterogeneity. Our study was likely insufficiently powered to detect differences in mortality, although there are other possible reasons for our failure to detect a mortality signal, such as confounding by severity of illness or therapeutic context. Based on our observations here, future studies in this field will likely require 750–1500 patients to exclude an association between strain and mortality, depending on the distribution of covariates.

Our study differs from other echocardiographic studies of septic patients in that the echoes were performed very early in the course of sepsis. Several other studies of septic patients performed echocardiography much later, around 24–48 h after the sepsis onset [19, 65–67]. Cardiac dysfunction evolves over the course of sepsis resuscitation [68], and our findings may not generalize to later sepsis. In our study, patients had all undergone initial volume resuscitation (median intravenous volume expansion 3 L) preceding the echocardiogram. Additionally, 39% were receiving vasopressors, which in themselves can negatively affect ventriculoarterial coupling and worsen ventricular function [68–70]. We did not have the data necessary to calculate ventriculoarterial uncoupling, which may be relevant to cardiac dysfunction in sepsis. We observed that patients with higher preload had received less intravenous fluid prior to the echo. We hypothesize that patients with higher preload may not have responded to fluid resuscitation, resulting in clinicians adopting a fluid-conservative resuscitation strategy, while low-preload patients have received multiple volume expansions based on positive response to fluid.

The observed association between tachycardia and LV systolic dysfunction can be explained by multiple mechanisms. In low-preload states, tachycardia may be a compensatory response to reduced cardiac preload to maintain cardiac output [24]. However, in high-preload states, after initial therapy with intravenous volume expansion, high adrenergic tone may induce persistence of tachycardia [71] and contribute to cardiac dysfunction [57] while increasing myocardial oxygen consumption and decreasing diastolic filling and coronary perfusion [72, 73]. Our observation of decreased myocardial function in the setting of tachycardia *and* high preload supports this model and is the main contribution of this study.

It is possible that profound vasoplegia and its treatment with vasoactive medications might be driving septic myocardial dysfunction [68, 74], although vasopressor dosage was not significant in our regression models. In such patients, there may be value in examining associations between left ventricular hyperkinesis (LVEF >75%), strain, and preload. However, we only observed 26 patients with LV hyperkinesis, limiting inferences in this population. We acknowledge that tachycardia in the absence of hypovolemia may result from pain, anemia, electrical conduction abnormalities, and hyperthyroidism. While we were unable to measure pain, no patient had concomitant thyroid storm. Although 23% of study patients had a hemoglobin <7 g/dL at some point within 24 h of the echo, all study ICUs typically transfuse to maintain hemoglobin >7 g/dL as a matter of practice.

One notable feature of tachycardia in sepsis is that the observed myocardial dysfunction may arise from a perturbation in the force–frequency and frequency-dependent acceleration of relaxation (FDAR) mechanisms, whereby tachycardia my worsen contractility due to decreased reuptake of calcium in the sarcoplasmic reticulum [59]. This mechanism may operate in parallel with toxic effects of hyperadrenergia. In other words, the association between tachycardia and impaired systolic function may reflect both direct effects of heart rate and common effects of high adrenergic tone.

While marked by a large sample size and echocardiography performed during the early phase of sepsis most relevant to therapeutic interventions, our study nevertheless has limitations. Our definition of cardiac preload on the basis of LV diastolic filling patterns is an imperfect surrogate for left ventricular end-diastolic volume. *E*/*e*′ may be more representative of ventricular elastance than ventricular volumes per se. However, *E*/*e*′ has been demonstrated to correlate well with left ventricular end-diastolic pressures and can be easily obtained in most patients with interpretable echo images [41, 47, 48]. Our definitions for severe sepsis and septic shock [37], although appropriate at the time of study and used in recent large trials of sepsis [38–40], have subsequently been replaced by the Sepsis-3 criteria [45]. Although 78% percent of patients in this study also met Sepsis-3 criteria, we lack information on non-enrolled patients who might have also met Sepsis-3 criteria. Therefore, this cohort of patients may not precisely match patients with sepsis defined by the new criteria. Patients in septic shock were receiving vasopressor infusions, which can worsen myocardial dysfunction, tachycardia, and strain [16, 68]. We did, however, adjust for vasopressor infusion rates in our analyses. About a third of eligible patients were excluded due to echocardiographic image quality, a well-known challenge in the critical care setting [41]. Importantly, our cohort compares favorably with other clinical cohorts in critical care in terms of the proportion of interpretable echoes [20, 46, 60]. The study may be insufficiently powered to detect a relationship between strain and tachycardia in low-preload states, as considerably fewer patients had low preload. Current guidelines advocate early volume expansion, and it is possible that we might see a different distribution of cardiac preload if this study were conducted at later time in the course of resuscitation. Strengths of this multicenter study include its relatively large size, the capture of echocardiographic data early in the course of sepsis (2.3 h after ICU admission, on average), and that interpreters were blinded to clinical outcomes and read the strain and *E*/*e*′ components of the echo separately. The inclusion of both patients receiving and not receiving mechanical ventilation and vasopressor infusions also increases the generalizability of the study.

## Conclusion

Tachycardia is associated with impaired LV strain, a sensitive marker of cardiomyopathy, in septic patients with high cardiac preload.

## Additional file

**Additional file 1: Table S1.** Patients stratified according to presence of shock (presence of vasopressor at time of echo) or no shock.

## Abbreviations

APACHE II: Acute Physiology and Chronic Health Evaluation, 2nd version
*E*/*e*′: ratio of early diastolic septal mitral inflow velocity to early diastolic mitral annulus velocity
FDAR: frequency-dependent acceleration of relaxation
ICU: intensive care unit
LV: left ventricle
LVEF: left ventricular ejection fraction
SOFA: Sequential Organ Failure Assessment
TTE: transthoracic echocardiography
