# Supplementary material for: Associations among left ventricular systolic function, tachycardia, and cardiac preload in septic patients
Source: Ann Intensive Care. 2017 Feb 17;7:17. doi: 10.1186/s13613-017-0240-2 (PMC5315651; doi:10.1186/s13613-017-0240-2)
Supplement: Supplementary file 1 — Additional file 1: Table S1. Patients stratified according to presence of shock (presence of vasopressor at time of echo) or no shock. [file 13613_2017_240_MOESM1_ESM.docx]

Table S1. Patients stratified according to presence of shock (presence of vasopressor at time of echo) or no shock.

| Variable  [Median (IQR) or N (%)] | Shock  (N=175) | Without Shock (N=277) | p-value |
| --- | --- | --- | --- |
| Cohort Details |  |  |  |
| Female | 90 (51%) | 150 (54%) | 0.56 |
| Age, years | 63 (51 - 73) | 67 (55 - 76) | 0.05 |
| Body Mass Index | 28 (24 - 34) | 28 (23 - 33) | 0.35 |
| APACHE II | 28 (21 - 34) | 23 (17 - 30) | **<0.001** |
| SOFA | 11 (8 - 13) | 8 (6 - 11) | **<0.001** |
| MAP, mm Hg | 68 (64 - 76) | 69 (59 - 77) | 0.31 |
| Mechanically ventilated | 83 (47%) | 55 (20%) | **<0.001** |
| PiO_2_/FiO_2_ Ratio, mm Hg | 223 (153 - 302) | 242 (166 - 376) | 0.07 |
| Serum lactate, mmol/dL | 2.3 (1.4 - 3.8) | 2.3 (1.4 - 3.8) | 0.84 |
| Heart rate, BPM | 99 (82 - 111) | 95 (83 - 112) | 0.88 |
| Fluid (6h prior to echo), mL** | 3000 (1230 - 4700) | 2850 (1210 - 4000) | 0.23 |
| Echocardiographic Parameters |  |  |  |
| Ejection Fraction, % | 61 (52 - 70) | 61 (52 - 68) | 0.22 |
| Strain, % | -16 (-21 - -12) | -17 (-20 - -13) | 0.93 |
| E/e' | 10.6 (8.3 - 13.9) | 13.2 (9.6 - 18.6) | **<0.001** |
| Stroke Volume, mL | 54 (44 - 73) | 58 (45 - 70) | 0.79 |
| Diastolic Function* |  |  | **0.01** |
| Grade 0 (Normal) | 82 (59%) | 115 (44%) |  |
| Grade 1 | 11 (8%) | 12 (5%) |  |
| Grade 2 | 11 (8%) | 32 (12%) |  |
| Grade 3 | 3 (2%) | 13 (5%) |  |
| Indeterminate | 31 (22%) | 88 (34%) |  |
| Clinical Outcomes |  |  |  |
| Inpatient Mortality | 41 (23%) | 46 (17%) | 0.09 |
| 28-day Mortality | 47 (27%) | 56 (20%) | 0.11 |
| OFFD Cardiovascular to day 14 | 12 (6 - 13) | 13 (12 - 13) | **<0.001** |
| OFFD Coagulation to day 14 | 14 (11 - 14) | 14 (13 - 14) | 0.06 |
| OFFD Hepatic to day 14 | 14 (10 - 14) | 14 (13 - 14) | **0.025** |
| OFFD Renal to day 14 | 13 (7 - 14) | 13 (13 - 14) | **0.011** |

*These data were only collected in the 398 patients from Intermountain ICUs, 138 with shock and 260 without Shock.

APACHE: Acute physiology and Chronic Health Evaluation; SOFA: Sequential Organ Failure Assessment; MAP: Mean Arterial Pressure; E/e’: ratio of early diastolic mitral inflow blood velocity to early diastolic mitral annular tissue velocity. OFFD: Organ-Failure-Free Days.
